# Construction of Bi-Enzyme Self-Assembly Clusters Based on SpyCatcher/SpyTag for the Efficient Biosynthesis of (R)-Ethyl 2-hydroxy-4-phenylbutyrate

**DOI:** 10.3390/biom13010091

**Published:** 2023-01-01

**Authors:** Jinmei Wang, Yuan Lu, Pengpeng Cheng, Chuyue Zhang, Lan Tang, Lihua Du, Jinghua Li, Zhimin Ou

**Affiliations:** College of Pharmaceutical Science, Zhejiang University of Technology, Hangzhou 310014, China

**Keywords:** carbonyl reductase CpCR, SpyCatcher/SpyTag, multi-enzyme cascade reactions, bi-enzyme self-assembly clusters (BESCs), (*R*)-HPBE

## Abstract

Cascade reactions catalyzed by multi-enzyme systems are important in science and industry and can be used to synthesize drugs and nutrients. In this study, two types of macromolecules of bi-enzyme self-assembly clusters (BESCs) consisting of carbonyl reductase (CpCR) and glucose dehydrogenase (GDH) were examined. Stereoselective CpCR and GDH were successfully fused with SpyCatcher and SpyTag, respectively, to obtain four enzyme modules, namely: SpyCatcher-CpCR, SpyCatcher-GDH, SpyTag-CpCR, and SpyTag-GDH, which were covalently coupled in vitro to form two types of hydrogel-like BESCs: CpCR-SpyCatcher-SpyTag-GDH and GDH-SpyCatcher-SpyTag-CpCR. CpCR-SpyCatcher-SpyTag-GDH showed a better activity and efficiently converted ethyl 2-oxo-4-phenylbutyrate (OPBE) to ethyl(R)2-hydroxy-4-phenylbutanoate ((*R*)-HPBE), while regenerating NADPH. At 30 °C and pH 7, the conversion rate of OPBE with CpCR-SpyCatcher-SpyTag-GDH as a catalyst reached 99.9%, with the *ee*% of (*R*)-HPBE reaching above 99.9%. This conversion rate was 2.4 times higher than that obtained with the free bi-enzyme. The pH tolerance and temperature stability of the BESCs were also improved compared with those of the free enzymes. In conclusion, bi-enzyme assemblies were docked using SpyCatcher/SpyTag to produce BESCs with a special structure and excellent catalytic activity, improving the catalytic efficiency of the enzyme.

## 1. Introduction

Chiral alcohols are widely used in the production of various chemicals and pharmaceuticals. Ethyl (R)-2-hydroxy-4-phenylbutanoate ((*R*)-HPBE) is an important chiral alcohol intermediate for the synthesis of angiotensin-converting enzyme inhibitors such as enalapril and lisinopril. Various methods for preparing (*R*)-HPBE have been developed, including multistep chemical synthesis [1], asymmetric enzymatic reduction of 2-oxo-4-phenyl-butyric acid ethyl ester (OPBE), kinetic resolution of racemates, and the enzymatic esterification of 2-hydroxy-4-phenylbutanoic acid [2,3,4]. Asymmetric catalysis is one of the most efficient methods to prepare (*R*)-HPBE [5,6]. In recent years, the asymmetric reduction of OPBE to (*R*)-HPBE using recombinant carbonyl reductase as a catalyst has attracted much attention because of its advantages of having a high yield, mild reaction conditions, green environmental protection, and economic feasibility [7]. However, the reaction catalyzed by carbonyl reductase is limited by cofactor recycling. To enhance the regeneration of cofactors in the biosynthesis of chiral alcohols, the transformation efficiency can be improved using multi-enzyme systems, substrate coupling, and enzyme coupling techniques [8,9,10]. In this study, the cofactor NADPH was externally added as a hydrogen donor for the carbonyl reductase-mediated asymmetric reduction of OPBE to promote conversion efficiency and affect the conversion of reduction. To reduce costs and improve the catalytic efficiency, glucose dehydrogenase (GDH) will be employed to regenerate NADPH.

The metabolic pathway formed by a multi-enzyme cascade is of great significance for the normal physiological metabolism of organisms and for the preparation of essential metabolites [11]. Multi-enzyme complex systems have become a hotspot in biological research because of their high catalytic capacity. Therefore, the construction of efficient and stable multi-enzyme cascades has a broad application prospect in the field of biocatalysis [12,13]. Advances in synthetic and systems biology have provided new ideas for biocatalysis and new methods for designing cascade reactions [14]. The cascade reaction catalyzed by several enzymes allows for the interaction of enzymes with different functions in the same catalytic reaction system so that various complex chemical reactions can be completed in one step [15]. The advantages of biological multi-enzyme cascade reactions include an improved reaction efficiency, no need to separate the reaction intermediates, and being eco-friendly. In a cascade reaction, the product of one enzyme is the substrate of another enzyme [16,17]. However, designing new bi-enzyme self-assembly clusters (BESCs) is challenging. The design of efficient BESCs requires the consideration of several factors that may affect the catalyst and cascade performance, such as the reaction conditions for each biocatalyst, the chemical equilibrium of the enzyme activity, and the stability of the enzyme [18].

In nature, cellular enzymes work in concert with metabolic pathways. These enzymes are organized in a temporal and spatial manner and play key roles in catalyzing different reactions [19,20,21,22]. Examples of this include pyruvate dehydrogenase complexes, polyketide synthase, and type I fatty acid synthase (FAS) [23,24,25]. The spatial organization of enzymes can combine two or more enzymatic steps into a biocatalytic cascade sequence to construct a stable and efficient cell-free in vitro biocatalytic multi-enzyme cascade [11,26]. As a classic example, Fontes et al. used modular dockerin–cohesin interactions to assemble cellulolytic enzymes on a scaffold protein backbone to form a nanomachine called a cellulosome, which can break down cellulose and hemicellulose in plant cell walls [27,28,29]. Zhang et al. assembled polyphosphate kinase (PPK) and bifunctional glutathione synthetase (GshF) to construct supramolecular nanoreactors for in vitro adenosine triphosphate (ATP) regeneration, where multi-enzyme nanoreactors (MENRs) were applied to an in vitro dual-enzyme cascade reaction. This cascade reaction system regenerated ATP 57% more efficiently than the unassembled enzyme mixture [30]. These examples show that cascade reactors formed by multi-enzyme assemblies can enhance the reaction efficiency.

**Figure 1:**
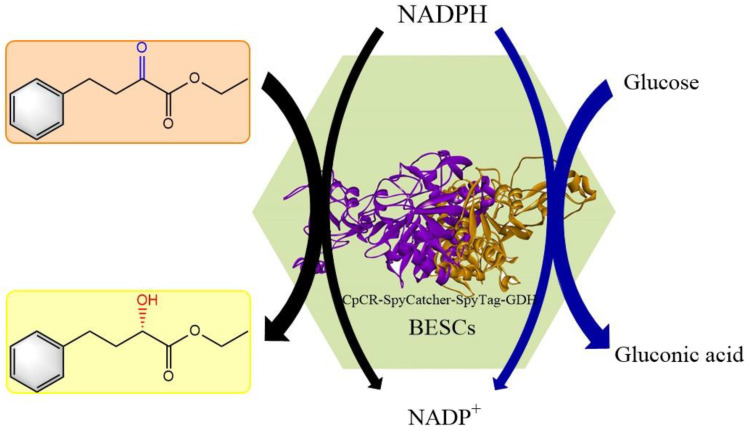
The biosynthesis mechanism of R-HPBE by BESCs. Enzymatic cascade driven by the two biocatalysts.

Recently, multi-enzyme cascade reactions have gained immense interest in the field of biotransformation. To date, there have been three main strategies for constructing multi-enzyme structures: fusion proteins, immobilization, and enzyme scaffold complexes [31]. The SpyCatcher/SpyTag system, derived from the CnaB2 structural domain, can form stable isopeptide bonds in various conditions, where the Lys on SpyCatcher and the Asp on SpyTag can spontaneously form covalent bonds. Therefore, the SpyCatcher/SpyTag system has become an important new technology for multi-enzyme applications [32,33,34]. In this study, we constructed two in vitro BESCs, CpCR-SpyCatcher-SpyTag-GDH and GDH-SpyCatcher-SpyTag-CpCR, by fusing SpyCatcher and SpyTag with carbonyl reductase (CpCR) and glucose dehydrogenase (GDH), respectively. These macromolecular bi-enzyme clusters have an excellent catalytic performance, which is conducive to the biosynthesis of drug intermediates, and can be applied to the biosynthesis of (*R*)-HPBE using the coenzyme regeneration system. The biosynthesis mechanism of (*R*)-HPBE is shown in Figure 1. Optimal BESCs with a higher activity were selected to compare the catalytic activities of the two types of BESCs. Furthermore, the structure and morphology of the optimized BESCs were analyzed using a field emission scanning electron microscope (FE-SEM), transmission electron microscopy (TEM), atomic force microscopy (AFM), and Fourier transformed infrared (FTIR).

## 2. Materials and Methods

### 2.1. Materials

ATCC 7330 was purchased from the American Typical Culture Preservation Center. The gene for *Bacillus subtilis* GDH, SpyCatcher sequence with a (GGGGS)_2_ short peptide linker and a linker-(AGAGAGPEG)_5_ were synthesized by Tsingke (Suzhou, China). Restriction endonucleases were purchased from TaKaRa Bio (Dalian, China). Polymerase chain reaction (PCR) reagents were purchased from Yeasen (Shanghai, China). All of the other chemicals and reagents were of analytical grade (Sangon, Shanghai, China).

### 2.2. Bacterial Strains and Plasmids

*Escherichia coli* DH5α was used as the host cell for plasmid amplification, while *E. coli* BL21 (DE3) was used as the host cell for the recombinant protein expression (Table 1). Luria–Bertani (LB) medium was used for the *E. coli* cell culture and fermentation. The concentration of antibiotics used for *E. coli* culture was 100 mg/L ampicillin or 50 mg/L kanamycin. In this study, pETDuet-1 and pET28a(+) plasmids were used as vectors for expression.

### 2.3. Construction of CpCR and GDH Recombinant Strains

The sequences of all of the PCR primers used in this experiment are listed in Table 2. The plasmids are summarized in Table 3, and the sequences of all of the proteins in this study are listed in Table 4. The *cpcr* gene was obtained via PCR amplification using the primers CpCR-F1 and CpCR-R1, using the whole genome of ATCC 7330 as a template. The *cpcr*-His gene (*cpcr* with a 6×His-Tag) was obtained via PCR amplification using the primers CpCR-F1 and CpCR-R2, using the whole genome of ATCC 7330 as a template. The plasmids pETDuet-1, pETDuet-SpyCatcher-(GGGGS)_2_, and the amplified products cpcr and cpcr-His were digested with restriction enzymes *Pst*I and *Xho*I, and then ligated with T4 DNA ligase to obtain pETDuet-CpCR and pETDuet-SpyCatcher-CpCR, respectively. The plasmids pET28a-SpyTag-(AGAGAGAGPEG)_5_ and pET28a-GDH were digested with the restriction enzymes *Bam*HI and *Xho*I to obtain pET28a-SpyTag-(AGAGAGAGPEG)_5_ and *GDH* genes, respectively. T4 DNA ligase was then used to obtain pET28a-Tag-GDH. The plasmids pET28a-Tag-CpCR and pETDuet-Catcher-GDH were constructed using seamless cloning techniques. In addition, pETDuet-CpCR, pETDuet-SpyCatcher-CpCR, pET28a-Tag-CpCR, pET28a- GDH, pET28a-Tag-GDH, and pETDuet-Catcher-GDH plasmids were constructed, the sequences of which were verified via sequencing. The constructed plasmids were then transformed into *E. coli* BL21 (DE3) cells for recombinant protein expression.

### 2.4. Protein Expression and Purification

The recombinant strains BL21-pETDuet-SpyCatcher-CpCR and BL21-pETDuet-SpyCatcher-GDH were inoculated in LB medium containing 100 µg/mL ampicillin, whereas BL21-pET28a-SpyTag-CpCR and BL21-pET28a-SpyTag-GDH were inoculated into LB containing 50 µg/mL kanamycin and were cultured at 37 °C. When the OD_600_ reached 0.6–0.8, 0.5 mM IPTG was added to the culture and induction was performed at 18 °C for 16 h. The culture was then centrifuged at 9000 rpm for 10 min to collect the cells, and the pellet was washed three times with 0.9% (*w*/*v*) saline. The bacterial pellet collected was then suspended in 100 mM phosphate buffer (PB) at pH 8.0, and the cells were lysed using an ultrasonic cell breaker. The bacterial lysate was centrifuged at 12,000 rpm for 10 min at 4 °C, and the supernatant was collected and filtered through a water-based microporous membrane (0.22 µm) to obtain a crude enzyme solution. CpCR, SpyCatcher-CpCR, SpyTag-CpCR, GDH, SpyTag-GDH, and SpyCatcher-GDH were purified via affinity chromatography using a BeyoGold His-tag Purification Resin (Beyotime, Shanghai, China). The purified proteins were dialyzed against 10 mM PB (pH 8.0).

### 2.5. Enzyme Activity Assays

The enzymatic activity of CpCR, SpyCatcher-CpCR, and SpyTag-CpCR in the reduction of OPBE was determined through the spectrophotometric detection of NADPH depletion at 340 nm. The reaction mixture consisted of 100 mM PB (pH 7.0), 2.0 mM OPBE, and 0.1 mM NADPH. To detect the enzymatic activities of GDH, SpyTag-GDH, and SpyCatcher-GDH, the reaction mixture consisted of 100 mM PB (pH 7.0), 10 mM glucose, and 2.0 mM NADP^+^. All of the measurements were performed in triplicate at 30 °C for 1 min. An active unit was defined as the amount of enzyme required to catalyze 1 µmol of NADPH oxidation (CR) per minute or 1 µmol of NADP^+^ reduction (GDH) per minute. Protein concentrations were measured using the Bradford method with bovine serum albumin as a standard [35].

### 2.6. Construction of BESCs In Vitro

Self-assembly of BESCs (CpCR-SpyCatcher-SpyTag-GDH and GDH-SpyCatcher-SpyTag-CpCR) in vitro was performed by directly mixing the purified fusion enzymes SpyCatcher-CpCR and SpyTag-GDH, and SpyTag-CpCR and SpyCatcher-GDH. To explore the effect of the ratio of the individual modules on the protein assembly, the corresponding proteins (2 µM) were mixed at different ratios (1:3, 1:2, 1:1, 2:1, or 3:1) and fully assembled. At the end of each reaction, the samples were heated in an SDS loading buffer at 95 °C for 10 min. Protein separation was performed using SDS-PAGE with 12% polyacrylamide gels stained with Coomassie Blue.

### 2.7. Determination of the Optimal Bi-Enzyme Catalytic System

Nine reaction groups were established to explore the catalytic performance of BESCs and a free bi-enzyme mixture: (1) 25 µM CpCR and 25 µM GDH, (2) 25 µM CpCR and 25 µM SpyCatcher-GDH, (3) 25 µM CpCR and 25 µM SpyTag-GDH, (4) 25 µM SpyCatcher-CpCR and 25 µM GDH, (5) 25 µM SpyCatcher-CpCR and 25 µM SpyCatcher-GDH, (6) 25 µM CpCR-SpyCatcher-SpyTag-GDH (BESCs), (7) 25 µM SpyTag-CpCR and GDH, (8) 25 µM CpCR-SpyTag-SpyCatcher-GDH (BESCs), and (9) 25 µM SpyTag-CpCR and SpyTag-GDH. These reactions were performed at 25 °C and 180 rpm for 12 h, and the catalytic activities of the OPBE enzymes were compared.

### 2.8. Kinetic Analysis of BESCs

Three parallel groups were set up for each concentration with different mass concentrations of OPBE at 10, 20, 30, 40, and 50 mM as substrates to determine the kinetic parameters of the free enzymes and CpCR-SpyCatcher-SpyTag-GDH. The reactions were carried out at 30 °C and pH 7.0 for 8 min, and the absorbance values at OD_340_ were determined, respectively. The apparent kinetic constants Kcat and Km were obtained from the fitted curves using the Michaelis–Menten equation.

$$V_{0}=\frac{V_{max}\times \left[S\right]}{K_{m}+\left[S\right]}$$

### 2.9. Effect of the Assembly Time on the BESCs-Catalyzed Conversion of OPBE

The effect of th assembly time on the conversion of OPBE was investigated. SpyCatcher-CpCR (25 µM) and SpyTag-GDH (25 µM) were mixed in equal volumes and incubated for 1, 2, 3, 4, 5, and 6 h. The free enzyme used for the assembly was removed via ultrafiltration, and the conversion of each reaction system was measured. The conversion of free 25 µM CpCR and 25 µM GDH was used as a control.

### 2.10. Effect of pH and Temperature on BESCs-Catalyzed Conversion of OPBE

To determine the optimal pH, the reduction in OPBE by BESCs was assayed in 100 mM PB at different pH levels (5–9). The prepared BESCs were fully dissolved in buffers with different pH values at a final concentration of 25 µM. In addition, each reaction included 50 mM OPBE, 300 mM glucose, 0.1 mM NADP^+^, and 0.2 mL anhydrous ethanol, and was maintained at 25 °C for 12 h.

To determine the optimal reaction temperature, reduction reactions were carried out at 25, 30, 35, 40, and 45 °C. After each reaction, the supernatant was centrifuged and extracted thrice with ethyl acetate. The extract was dried with anhydrous magnesium sulfate for analysis, and the conversion and *ee*% of (*R*)-HPBE were determined via GC.

### 2.11. Dynamic Light Scattering (DLS) Assay of Optimal BESCs

The optimal BESCs were analyzed using Brookhaven Omni at an assay temperature of 25 °C. SpyCatcher-CpCR (2 µM), SpyTag-GDH (2 µM), and BESCs (2 µM) were added to the sample bath after sonication, and the particle sizes of the components were recorded.

### 2.12. FE-SEM, TEM, and AFM Analysis

For the FE-SEM, the optimal BESC samples were dried on a conductive adhesive and sprayed with a nanogold coating. Images were collected using a field emission-scanning electron microscope (HITACHI, Tokyo, Japan) at 10 kV. TEM images were obtained using a transmission electron microscope (HITACHI HT7700 EXALENS). For the AFM, the samples were added dropwise to mica sheets, dried, and observed using an AFM/Multimode Nanoscope (Bruker, Massachusetts, Germany, Dimension Icon).

### 2.13. Secondary Structure Analysis

Fourier transform infrared spectroscopy (FTIR) (Thermo Nicolet 6700, Thermo, MA, United States.) was performed on the optimized BESCs. The spectral measurement range was from wavenumbers between 4000 and 400 cm^−1^ at a resolution smaller than 0.09 cm^−1^.

### 2.14. Bioreduction of OPBE to (R)-HPBE

Here, 25 µM BESCs, 300 mM glucose, 0.1 mM NADP^+^, 50 mM OPBE, and 0.2 mL anhydrous ethanol were added into 2 mL PB buffer (100 mM, pH 7). The components were mixed well and incubated at 25 °C and 180 rpm for 12 h. At the end of the reaction, the supernatant was centrifuged and extracted thrice with ethyl acetate. The samples were dried over anhydrous magnesium sulfate for analysis. The conversion and *ee*% of (*R*)-HPBE were determined via GC.

### 2.15. Analytical Methods

The conversion and *ee*% of R-HPBE were analyzed using a Shimadzu GC-2014 gas chromatograph. “Conversion” represents the catalytic efficiency and was defined as the ratio of the amount of substrate converted to product to the initial amount of substrate. In this study, the samples were analyzed using a gas chromatograph equipped with an Agilent CP7502 J&W CP-ChirasilDex CB chiral column (Machery-Nagel; 25 m × 0.25 mm × 0.25 mm). The injector, column, and FID temperatures were 250, 130, and 250 °C, respectively. The split ratio was 1:15; the flow rate was 2 mL/min; and the retention times of OPBE, (*R*)-HPBE, and (S)-HPBE were 18.5, 25.5, and 26.5 min, respectively.

Equations (1) and (2) define the conversion and *ee*% of (*R*)-HPBE, as follows:

(1) $$X\left(\%\right)=\frac{M_{S}\times P}{M_{P}\times Q}\times 100\%$$

where *M_S_* is the molecular weight of the substrate, *M_P_* is the molecular weight of the product, *Q* is the mass of the substrate at the initial reaction, and *P* is the mass of the product at the end of the reaction, and

(2) $$ee\%=\frac{C_{R}-C_{S}}{C_{R}+C_{S}}\times 100\%$$

where *C_R_* and *C_S_* represent the concentrations of (*R*)-HPBE and (S)-HPBE, respectively.

## 3. Results and Discussion

### 3.1. Construction of CpCR, SpyCatcher-CpCR, SpyTag-CpCR, GDH, SpyCatcher-GDH, and SpyTag-GDH

**Figure 2:**
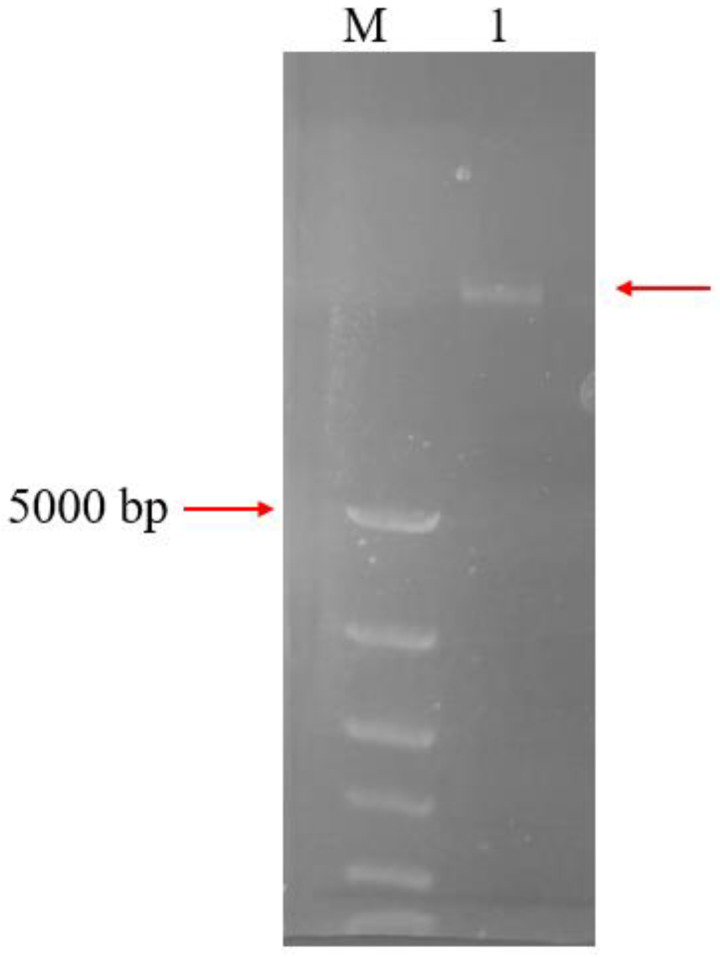
Agarose electrophoresis of the ATCC 7330 genome. Lane M: DNA marker. Lane 1: ATCC 7330 genome.

**Figure 3:**
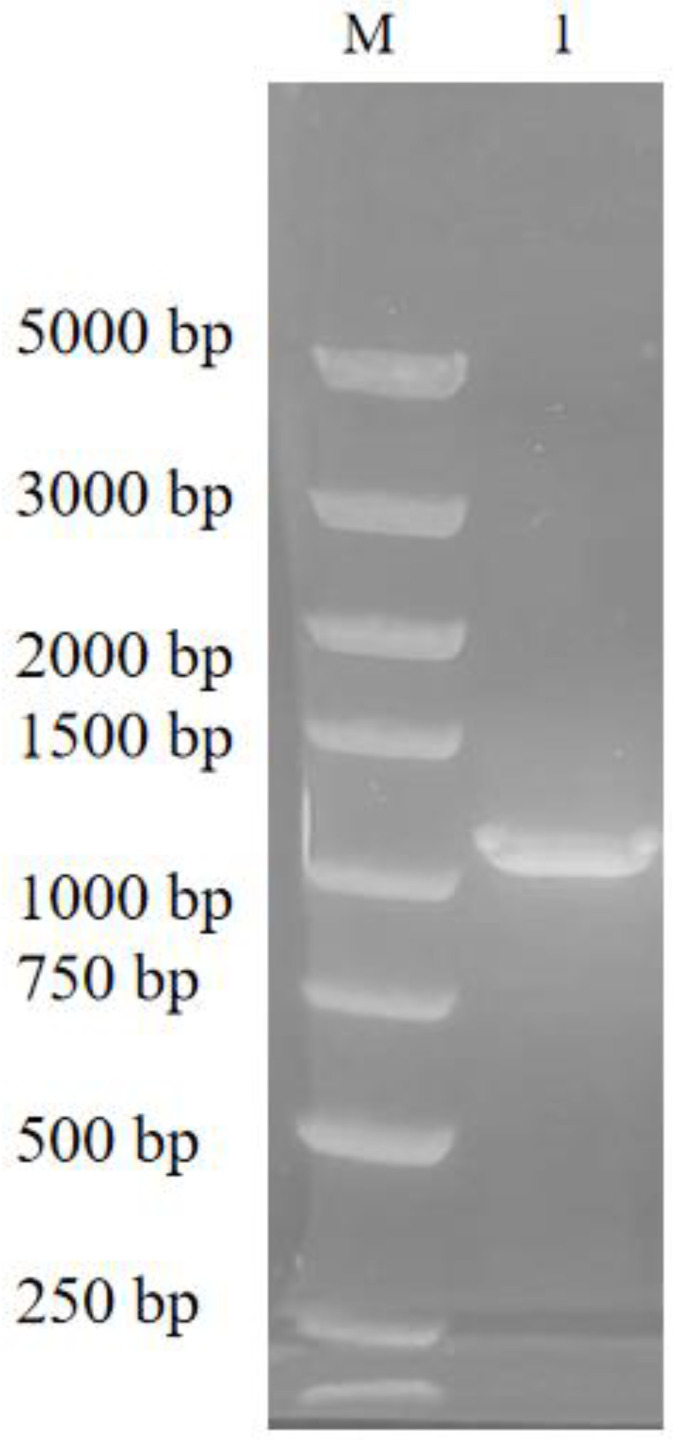
Agarose electrophoresis of colony PCR products. Lane M: DNA Marker. Lane 1: cpcr.

**Figure 4:**
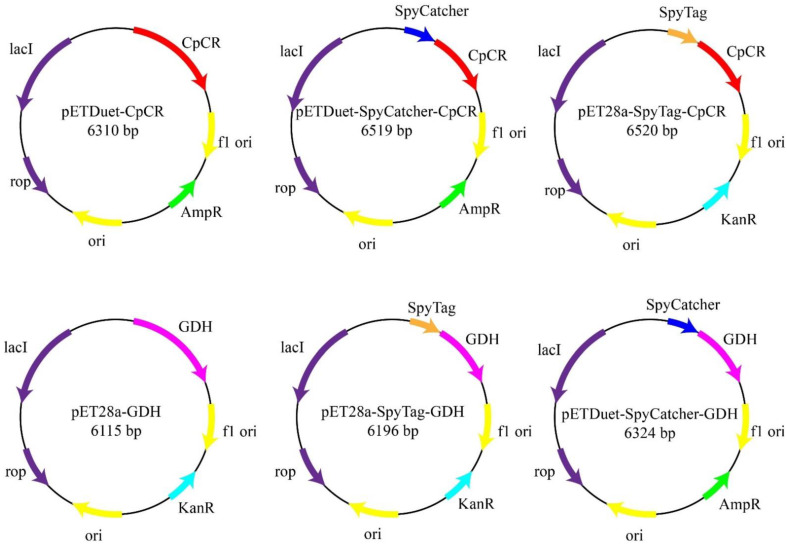
Construction of carbonyl reductase and glucose dehydrogenase fusion enzymes with SpyCatcher and SpyTag, respectively.

The chiral alcohol (*R*)-HPBE is an intermediate in the synthesis of lisinopril. *Candida parapsilosis* ATCC 7330 is a multifunctional catalyst with a broad substrate adaptability and enantioselectivity that can reduce OPBE to (*R*)-HPBE [36,37]. In this study, the CpCR gene was successfully cloned from *C. parapsilosis* ATCC 7330 after genomic DNA extraction (Figure 2) and PCR amplification (Figure 3), and was cloned and soluble-expressed in *E. coli*. Simultaneously, fusion enzyme plasmids of CpCR and GDH with SpyCatcher or SpyTag short peptides were also constructed (Figure 4).

**Figure 5:**
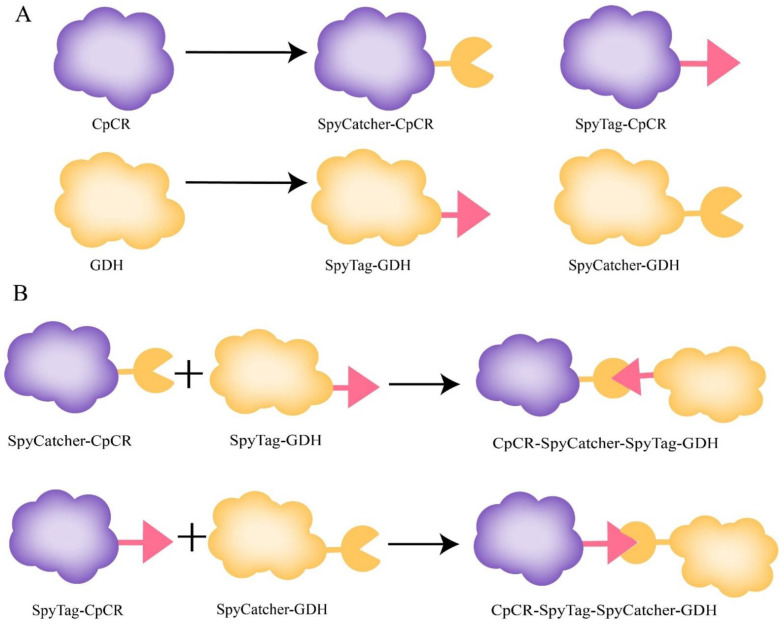
Spontaneous reaction between SpyCatcher- and SpyTag-fusion enzymes. (**A**) Six fusion enzymes. (**B**) Schematic diagram of spontaneous covalent reaction between purified target enzymes.

The SpyCatcher domain (~9.5 kDa) and SpyTag peptide (~1.5 kDa) were fused to the end of the model enzyme to automatically initiate cross-linking. To determine whether the fusion protein could be covalently bound, SpyCatcher and SpyTag were fused to the N-termini of CpCR and GDH to obtain SpyCatcher-CpCR, SpyCatcher-GDH, SpyTag-CpCR, and SpyTag-GDH. To ensure that the interacting proteins SpyCatcher and SpyTag could fully contact and weaken the interaction between adjacent protein structural domains, a segment of flexible linker 1 (GGGGS)_2_ was added after SpyCatcher, and a segment of flexible linker 2 (AGAGAGPEG)_5_ was added after SpyTag (Figure 5).

### 3.2. Expression and Purification of Recombinant Proteins

**Figure 6:**
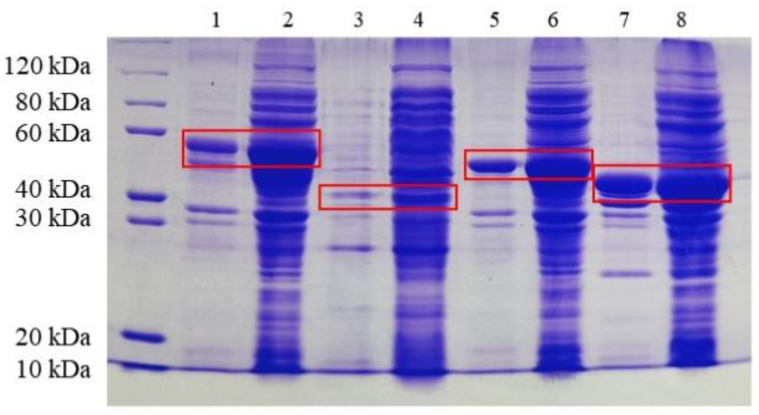
SDS-PAGE analysis of the expression of four recombinant fusion enzymes in *E. coli*. Lane 1 and 2: SpyCatcher-CpCR cell fragmentation pellet and supernatant; Lane 3 and 4: SpyTag-GDH cell fragmentation pellet and supernatant; Lane 5 and 6: SpyTag-CpCR cell fragmentation pellet and supernatant; Lane 7 and 8: SpyCatcher-GDH cell fragmentation pellet and supernatant.

The verified recombinant plasmid was introduced into the expression host *E. coli* BL21 (DE3). When the cell density (OD_600_) reached 0.8, the cultures of recombinant *E. coli* were induced with 0.5 mM IPTG. SDS-PAGE confirmed that the protein was expressed (Figure 6). The 6×His-tagged recombinant protein was subjected to His-tag affinity chromatography. Six purified proteins, CpCR (41 kDa), SpyCatcher-CpCR (54.5 kDa), SpyTag-CpCR (46.3 kDa), GDH (32 kDa), SpyCatcher-GDH (42.3 kDa), and SpyTag-GDH (34.1 kDa), were obtained as expected.

### 3.3. Analysis of the Enzymatic Activities of CpCR, SpyCatcher-CpCR, and SpyTag-CpCR

The enzyme activities of purified CpCR, SpyCatcher-CpCR, and SpyTag-CpCR were analyzed as described in Section 2.5. As shown in Table 5, CpCR showed a reducing activity of 16.67 U/mg, while the fusion enzyme SpyCatcher-CpCR modified by SpyCatcher showed little difference from the activity of CpCR. However, the expression of SpyTag-CpCR was significantly lower than that of CpCR and SpyCatcher-CpCR. It is likely that the binding of SpyTag to CpCR changed the conformation of the active center of CpCR, thereby decreasing the reducing activity of SpyTag-CpCR. In general, these three enzymes exhibited enzymatic activities at pH 7.0.

### 3.4. Analysis of the Enzymatic Activities of GDH, SpyCatcher-GDH, and SpyTag-GDH

The enzyme activities of purified GDH, SpyCatcher-GDH, and SpyTag-GDH were detected, as described in Section 2.5. Table 5 shows that the activity of the modified fusion enzyme was not significantly reduced compared to that of the unmodified GDH. In general, these three enzymes also exhibited enzymatic activities at pH 7.0.

### 3.5. Design and Preparation of BESCs

**Figure 7:**
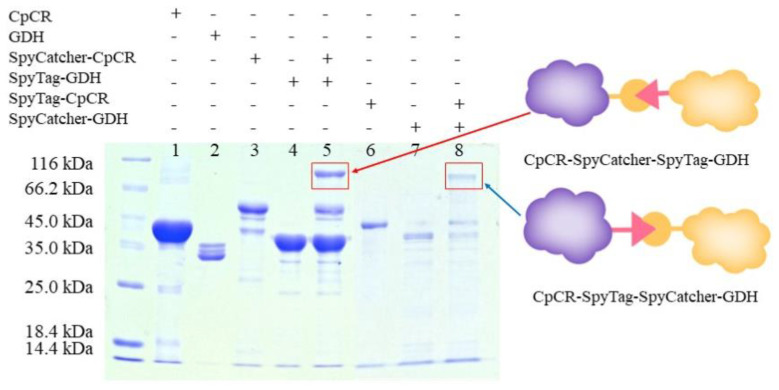
SDS-PAGE analysis of cross-linking reaction of fusion enzymes; Lane 1: CpCR (41 kDa); Lane 2: GDH (32 kDa); Lane 3: SpyCatcher-CpCR (54.5 kDa); Lane 4: SpyTag-GDH (34.1 kDa); Lane 5: CpCR-SpyCatcher-SpyTag-GDH(88.6 kDa); Lane 6: SpyTag-CpCR (46.3 kDa); Lane 7: SpyCatcher-GDH (42.3 kDa); Lane 8: CpCR-SpyTag-SpyCatcher-GDH(88.6 kDa).

The well-established SpyCatcher/SpyTag system, as a tool for protein–protein linkage, was used to construct BESCs. SpyCatcher and SpyTag spontaneously react to form covalent bonds, resulting in stable molecular self-assemblies. SpyCatcher-CpCR, SpyTag-GDH, SpyTag-CpCR, and SpyCatcher-GDH were mixed for covalent coupling reactions, as described in Section 2.6, and the products were detected using SDS-PAGE. Figure 7 shows that high-molecular-weight bands were produced in lanes 5 and 8. As expected, two BESCs were successfully assembled. Specifically, the bi-enzyme fusions CpCR-SpyCatcher-SpyTag-GDH (88.6 kDa) and CpCR-SpyTag-SpyCatcher-GDH (88.6 kDa) were successfully synthesized.

### 3.6. Determination of the Optimal Assembly Ratio for BESCs

**Figure 8:**
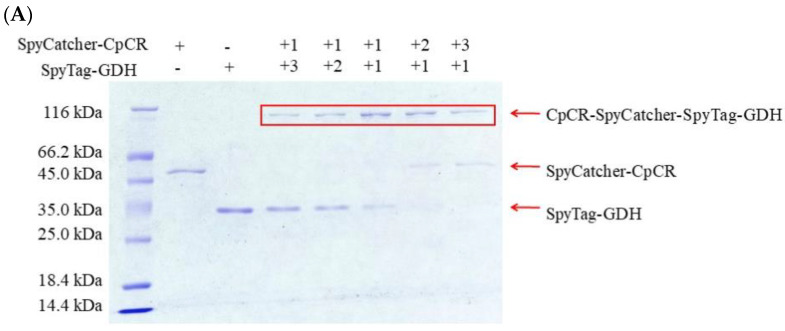

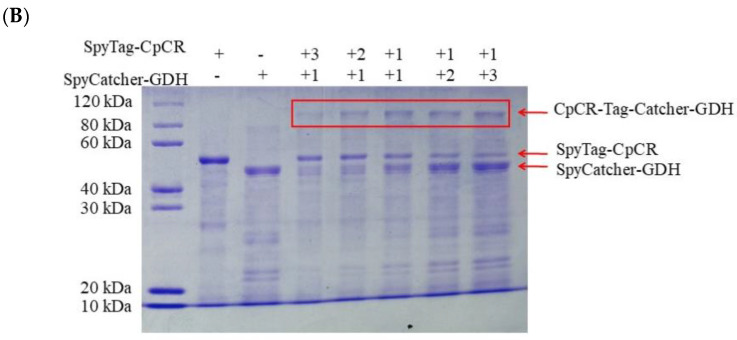
SDS-PAGE analysis of two groups of reactors assembled in different proportions. (**A**) SpyCatcher-CpCR and SpyTag-GDH assembled. (**B**) SpyTag-CpCR and SpyCatcher-GDH assembled.

Next, 2 µM each of SpyCatcher-CpCR and SpyTag-GDH and SpyTag-CpCR and SpyCatcher-GDH were mixed in different proportions, as per Section 2.6, to detect the optimal proportion of protein modules for BESCs assembly (Figure 8). The SDS-PAGE analysis showed that SpyCatcher and SpyTag formed isopeptide bonds and spontaneously formed covalent complexes of CpCR-SpyCatcher-SpyTag-GDH and CpCR-SpyTag-SpyCatcher-GDH, which were detected as bands of approximately 88.6 kDa in size. When CpCR-SpyCatcher-SpyTag-GDH was assembled, a small amount of SpyCatcher-CpCR or SpyTag-GDH could not be assembled even when their molar ratios were different. Similarly, when assembling CpCR-SpyTag-SpyCatcher-GDH, small amounts of SpyCatcher-GDH and SpyTag-CpCR remained. Subsequently, the protein concentration was measured after ultrafiltration (Table 6).

The assembly efficiency of CpCR-SpyCatcher-SpyTag-GDH was slightly lower than that of CpCR-SpyTag-SpyCatcher-GDH, which may be due to spatial site resistance during isopeptide bond formation. Therefore, a 1:1 molar ratio of CpCR-SpyCatcher and SpyTag-GDH was deemed the optimal value for assembly, with an assembly efficiency reaching 97.6%. For the subsequent experiments, a 1:1 molar ratio of SpyCatcher-CpCR to SpyTag-GDH was used to obtain BESCs.

### 3.7. Comparison of Catalytic Activity between BESCs and Free Enzymes

**Figure 9:**
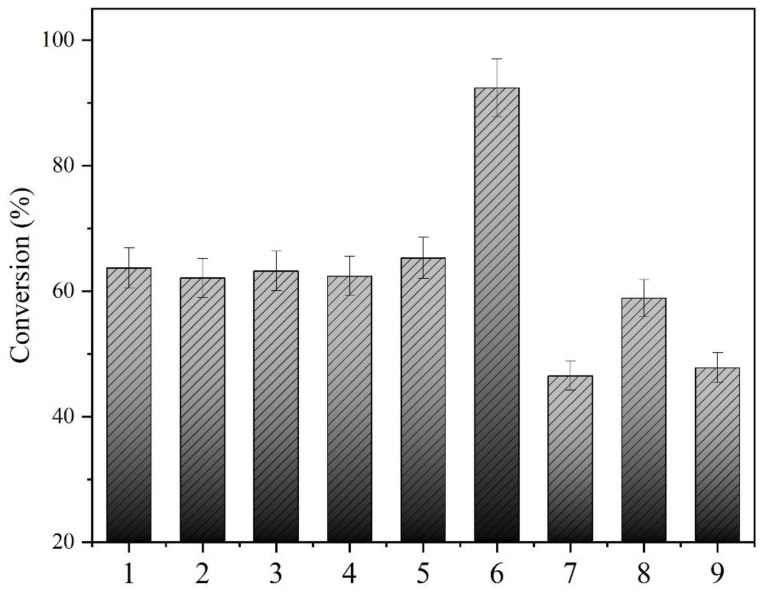
Comparison of activities of MESCs and free enzymes. (1) CpCR and GDH (free enzyme 1); (2) CpCR and SpyCatcher-GDH; (3) CpCR and SpyTag-GDH; (4) SpyCatcher-CpCR and GDH; (5) SpyCatcher-CpCR and SpyCatcher-GDH; (6) BESCs (CpCR-SpyCatcher-SpyTag-GDH); (7) SpyTag-CpCR and GDH; (8) BESCs (CpCR-SpyTag-SpyCatcher-GDH); (9) SpyTag-CpCR and SpyTag-GDH.

Nine reaction groups were established, as per Section 2.7, to examine the reduction of OPBE by BESCs. The consumption of OPBE and the generation of the product (*R*)-HPBE generation was quantified using GC. Figure 9 shows that the catalytic activity of the BESCs (CpCR-SpyCatcher-SpyTag-GDH) was 1.45-fold higher than that of the free enzyme (CpCR/GDH). In contrast, the catalytic activity of the BESCs (CpCR-SpyTag-SpyCatcher-GDH) was only 0.9 times that of the free enzyme (CpCR/GDH). Compared with the other uncoupled enzyme systems, the BESCs (CpCR-SpyCatcher-SpyTag-GDH) showed a higher catalytic activity.

Discovery Studio™ was used to simulate molecular docking. From the docking results of each enzyme with the OPBE molecule, CpCR, SpyCatcher-CpCR, SpyTag-CpCR, CpCR-SpyCatcher-SpyTag-GDH, and CpCR-SpyTag-SpyCatcher-GDH showed docking scores of 86.76, 90.78, 79.79, 83.68, and 76.78, respectively (Table 7).

**Figure 10:**
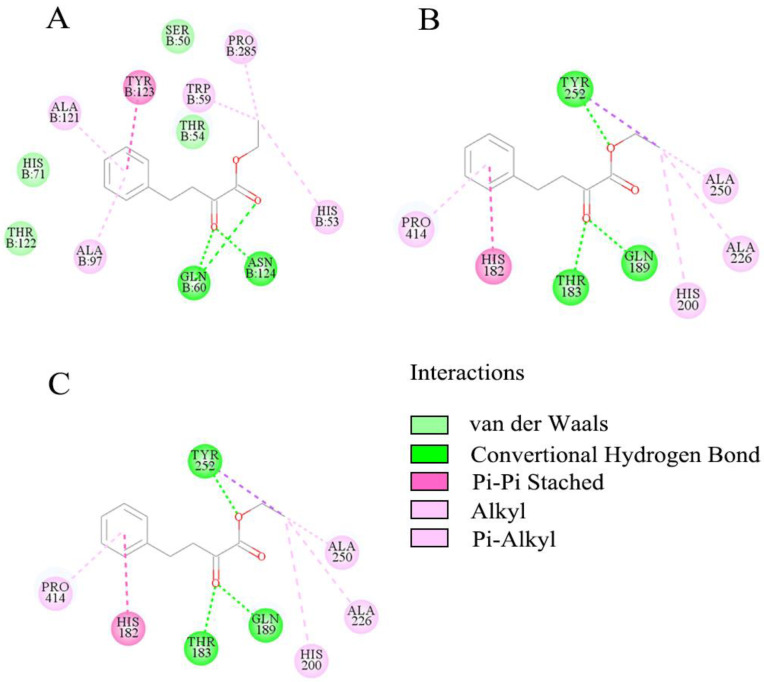
2D view of the docking results. (**A**) Docking of substrate OPBE into the active site of CpCR. (**B**) Docking of substrate OPBE into the active site of SpyCatcher-CpCR. (**C**) Docking of substrate OPBE into the active site of SpyTag-CpCR.

Figure 10 shows a two-dimensional plane diagram of the interaction between OPBE and each protein. Compared with CpCR and SpyCatcher-CpCR, the docking results of OPBE and SpyTag-CpCR allowed for a visualization of their interactions, in which the number of major amino acids and functional groups decreased in the molecular docking diagram. Comparing the performance of different enzymes, CpCR-SpyCatcher-SpyTag-GDH showed an optimum reduction activity and stereoselectivity, and was selected for further analyses.

### 3.8. The Reaction Kinetics Analysis of CpCR-SpyCatcher-SpyTag-GDH and Free Enzymes

Kinetic parameters are determined for the dual enzyme system in Table 8. Compared with the free enzymes, CpCR-SpyCatcher-SpyTag-GDH showed a higher catalytic efficiency. The Km, Kcat, and Kcat/Km values by the free enzymes were 23.94 mM, 1.76 s^−1^, and 0.074 s^−1^·mM^−1^ respectively. The Km values by CpCR-SpyCatcher-SpyTag-GDH were reduced by 9.15% compared with the free enzymes, suggesting that its affinity for the substrate OPBE may be improved, while the Kcat/Km value of CpCR-SpyCatcher-SpyTag-GDH was 1.31 times higher than that of the free enzymes.

### 3.9. Effect of Assembly Time on the Reducing Activity of CpCR-SpyCatcher-SpyTag-GDH

**Figure 11:**
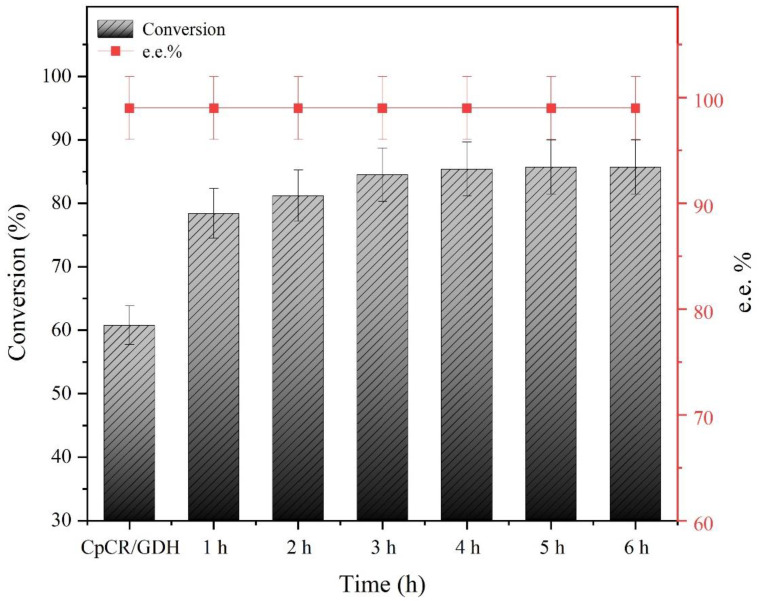
Effect of assembly time on CpCR-SpyCatcher-SpyTag-GDH.

In order to detect the effect of assembly time on the BESCs activity, 25 µM SpyCatcher-CpCR and 25 µM SpyTag-GDH were mixed at a 1:1 ratio and allowed to assemble at different times. The conversion of OPBE was analyzed at different assembly times for CpCR-SpyCatcher-SpyTag-GDH. The results showed that free SpyCatcher-CpCR and SpyTag-GDH rapidly completed the covalent coupling of proteins and that the conversion of OPBE by CpCR-SpyCatcher-SpyTag-GDH increased over time. The conversion rate stabilized after 3 h, reaching 84.5%, and the overall conversion increased by 1.4-fold compared with the free bi-enzyme (CpCR and GDH) system. Therefore, the optimal assembly time used for the subsequent experiments was 3 h. This result indicates that CpCR-SpyCatcher-SpyTag-GDH can effectively improve the cascade reaction compared with a mixture of two free enzymes (Figure 11). Moreover, the self-assembly time did not affect the enantiomeric excess of R-HPBE as the *ee*% of R-HPBE was maintained at 99% across the different assembly times.

### 3.10. Effect of pH on the Reducing Activity of CpCR-SpyCatcher-SpyTag-GDH

**Figure 12:**
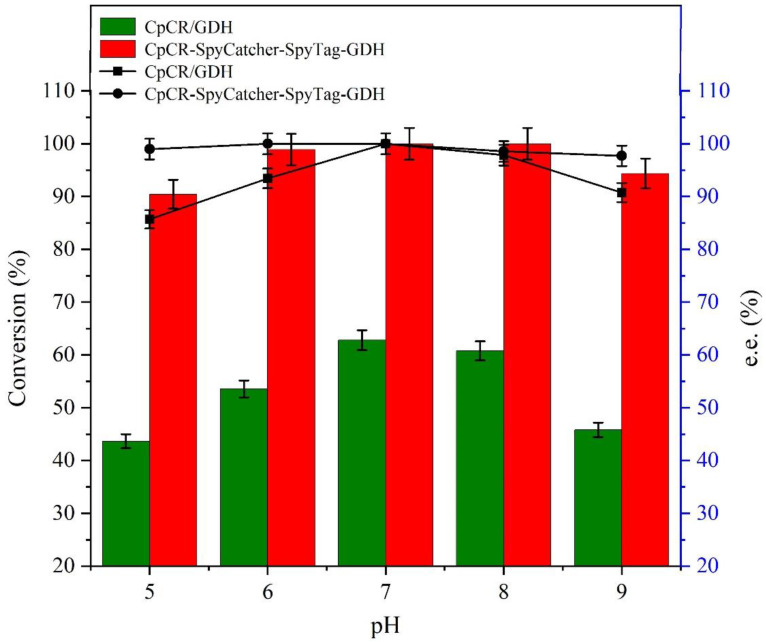
Effect of pH on CpCR-SpyCatcher-SpyTag-GDH and free enzymes.

The biocatalytic activity of CpCR-SpyCatcher-SpyTag-GDH was determined using the potential chiral ketone OPBE as the substrate. The stability of CpCR-SpyCatcher-SpyTag-GDH at different pH levels (pH 5–9) was investigated (Figure 12). Compared with the free bi-enzyme, the effect of pH on the catalytic activity of CpCR-SpyCatcher-SpyTag-GDH was not significant. CpCR-SpyCatcher-SpyTag-GDH maintained a high activity at pH 5–9, with a slight decrease in activity under acidic or basic environments. The catalytic activity of the free bi-enzyme system was affected by changes in pH. CpCR-SpyCatcher-SpyTag-GDH had the best catalytic activity at pH 7.0; conversion reached 99.9% and the *ee*% of (*R*)-HPBE was above 99.9%. The conversion by CpCR-SpyCatcher-SpyTag-GDH was 1.6 times higher than the free enzymes. The effect of pH on the *ee*% of (*R*)-HPBE by CpCR-SpyCatcher-SpyTag-GDH was significantly lower than that of the free bi-enzyme system. This may be due to the tight spatial structure formed by the self-assembly of the two enzymes in CpCR-SpyCatcher-SpyTag-GDH.

### 3.11. Effect of Temperature on the Reducing Activity of CpCR-SpyCatcher-SpyTag-GDH

**Figure 13:**
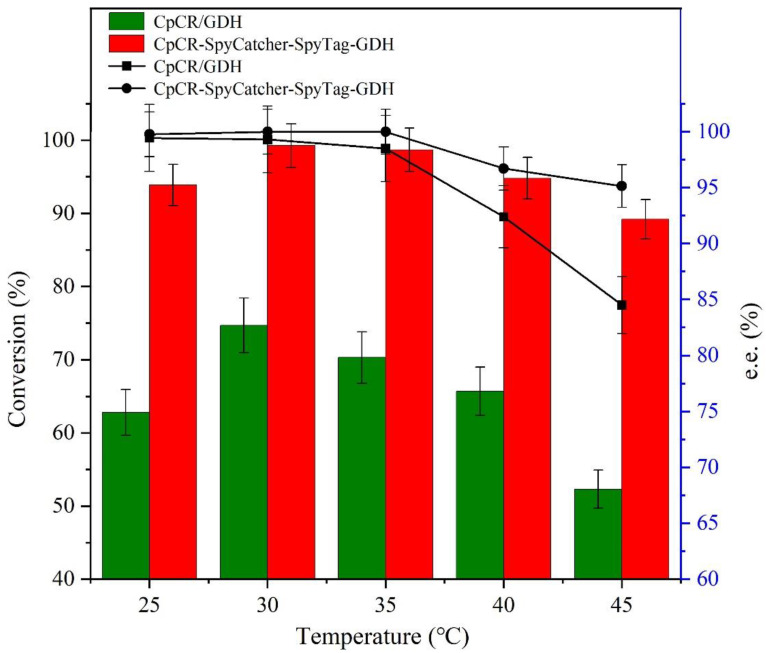
Effect of temperature on the activity of CpCR-SpyCatcher-SpyTag-GDH.

The stability of CpCR-SpyCatcher-SpyTag-GDH at different temperatures (25–45 °C) was investigated (Figure 13). CpCR-SpyCatcher-SpyTag-GDH had better temperature stability compared with the unassembled free bi-enzyme system. The higher the temperature, the better the relative stability of CpCR-SpyCatcher-SpyTag-GDH. The conversion of OPBE by CpCR-SpyCatcher-SpyTag-GDH was 1.5 times higher than that of the free enzyme at 25 °C and 1.7 times higher at 45 °C. At 30 °C, and the conversion of OPBE was the highest, reaching 99.9%. When the reaction temperature was below 35 °C, the temperature did not affect the *ee*% of (*R*)-HPBE by CpCR-SpyCatcher-SpyTag-GDH, as the *ee*% of (*R*)-HPBE was maintained at 99.9% across different temperatures. The temperature stability of CpCR-SpyCatcher-SpyTag-GDH was enhanced compared with that of the free bi-enzyme system, which may be due to the formation of a tight molecular structure after the BESCs assembly, which reduced the possibility of protein conformational changes.

### 3.12. Particle Size Determination of CpCR-SpyCatcher-SpyTag-GDH Using Dynamic Light Scattering (DLS)

**Figure 14:**
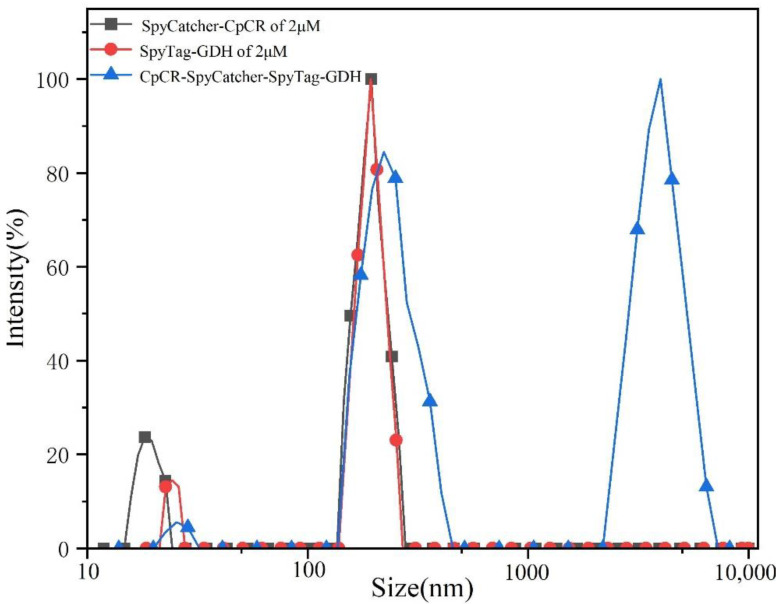
DLS analysis of CpCR-SpyCatcher-SpyTag-GDH.

DLS was used to measure the particle size of the CpCR-SpyCatcher-SpyTag-GDH. Figure 14 shows that the particle size of the unassembled free enzyme was mostly distributed in the 100–1000 nm range (>97%). Upon the assembly of CpCR-SpyCatcher-SpyTag-GDH, 5.3% of the enzyme molecules had particle sizes within the range of 100–1000 nm, and 94.6% of the molecules were cross-linked and converted to macromolecules. During the assembly process, several micrometer-sized assembly clusters appeared to form supramolecular bodies.

### 3.13. Structural Characterization of CpCR-SpyCatcher-SpyTag-GDH

**Figure 15:**
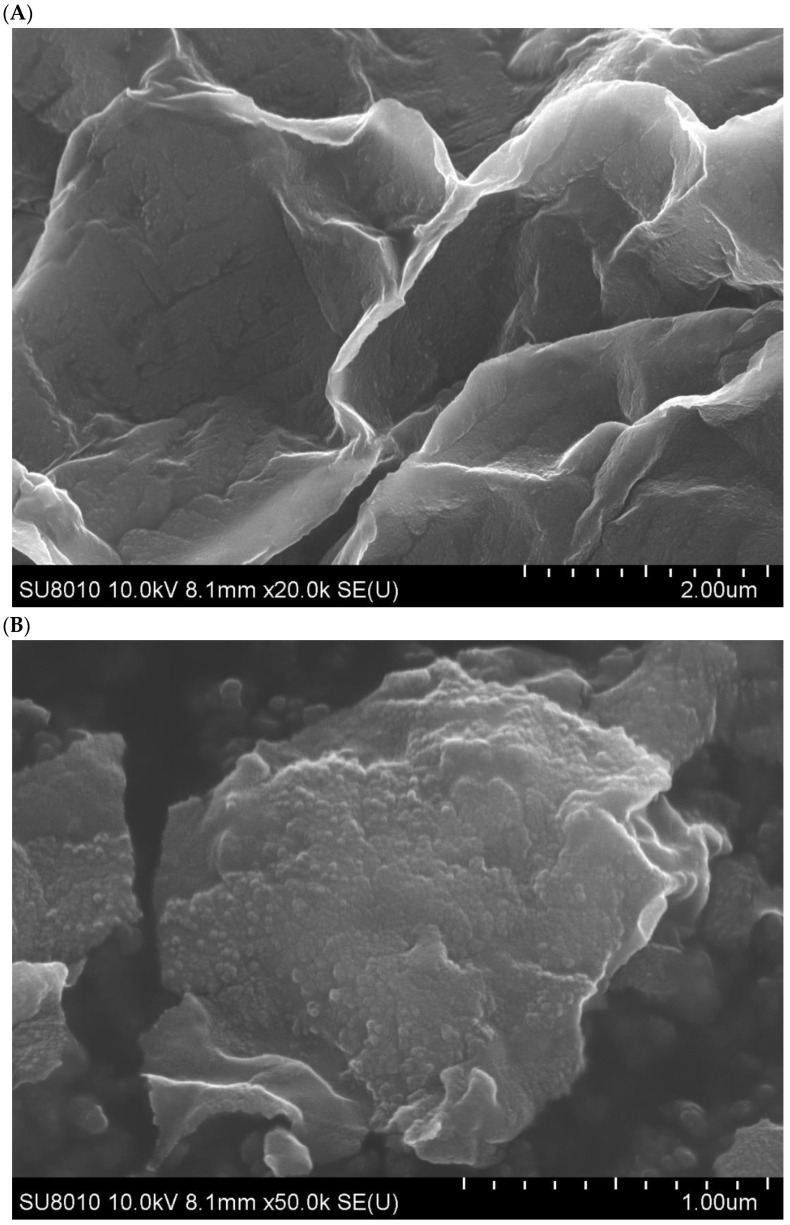
FE-SEM images of CpCR-SpyCatcher-SpyTag-GDH. (**A**) Morphological characterization of CpCR-SpyCatcher-SpyTag-GDH after magnification 20k times. (**B**) Morphological characterization of CpCR-SpyCatcher-SpyTag-GDH after magnification 50k times.

**Figure 16:**
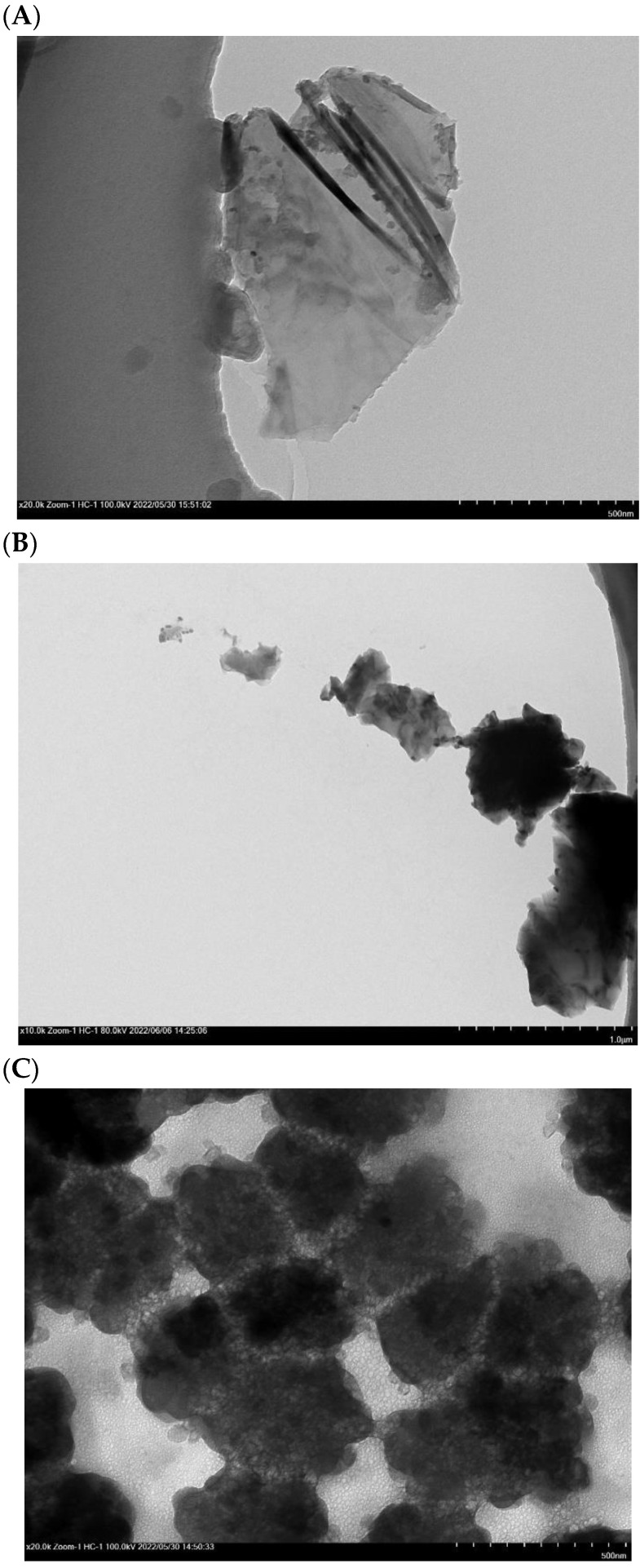
The representative TEM images. (**A**) SpyCatcher-CpCR. (**B**) SpyTag-GDH. (**C**) 10 k × CpCR-SpyCatcher-SpyTag-GDH.

FE-SEM, TEM, and AFM were used to examine the morphology of CpCR-SpyCatcher-SpyTag-GDH. The results showed that CpCR-SpyCatcher-SpyTag-GDH had a continuous two-dimensional sheet structure with sizes ranging from tens to hundreds of micrometers. The assemblies were folded and stacked to form a hydrogel-like structure (Figure 15). To further analyze the surface structure of CpCR-SpyCatcher-SpyTag-GDH, the assembly was subjected to high-resolution TEM. At 10,000× magnification, it can be observed that the surface of the assembly had a porous structure, which was different from SpyCatcher-CpCR and SpyTag-GDH before assembly. This result confirms that SpyCatcher-CpCR and SpyTag-GDH aggregate under the interaction of SpyCatcher and SpyTag to form a network structure (Figure 16).

**Figure 17:**
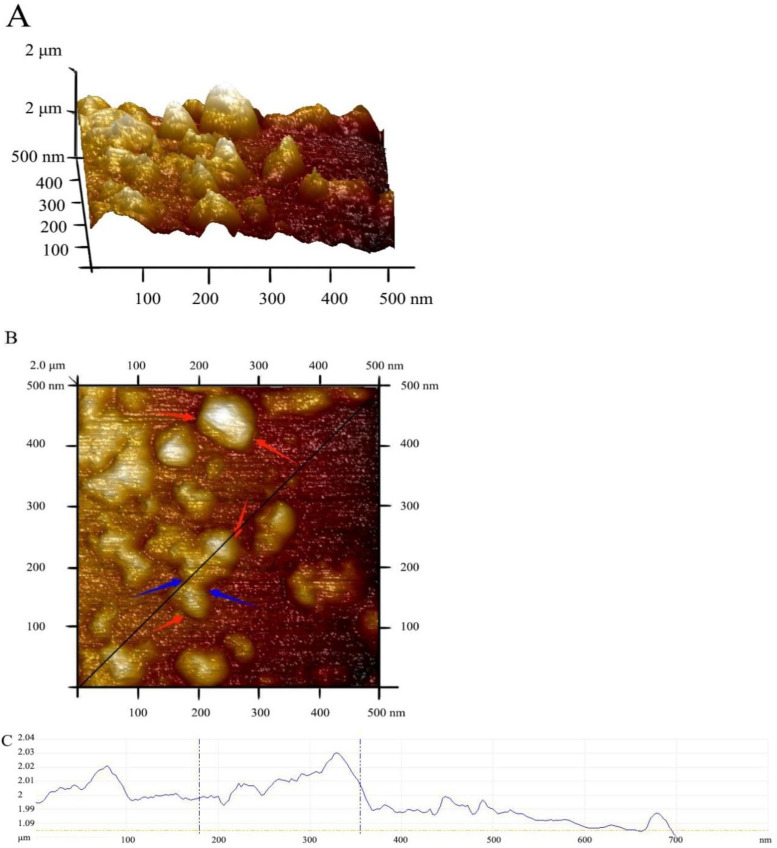
Surface topography of CpCR-SpyCatcher-SpyTag-GDH by AFM. (**A**) Side view by AFM. (**B**) Top view by AFM. (**C**) The cross-sectional analysis image. The red arrows indicate the boundaries of CpCR-SpyCatcher-SpyTag-GDH, the blue arrows indicate the intersecting and overlapping positions, and the uplift is formed by the aggregation and accumulation of CpCR-SpyCatcher-SpyTag-GDH.

The surface topography of CpCR-SpyCatcher-SpyTag-GDH was also analyzed using AFM (Figure 17). The cross-sectional image (C), shown by the black line in panel (B), indicates that CpCR-SpyCatcher-SpyTag-GDH was formed by the aggregation of particles with an average particle size of approximately 40 nm. The exemplary root-mean square roughness of this section was 3.14 nm.

### 3.14. Secondary Structure Analysis

**Figure 18:**
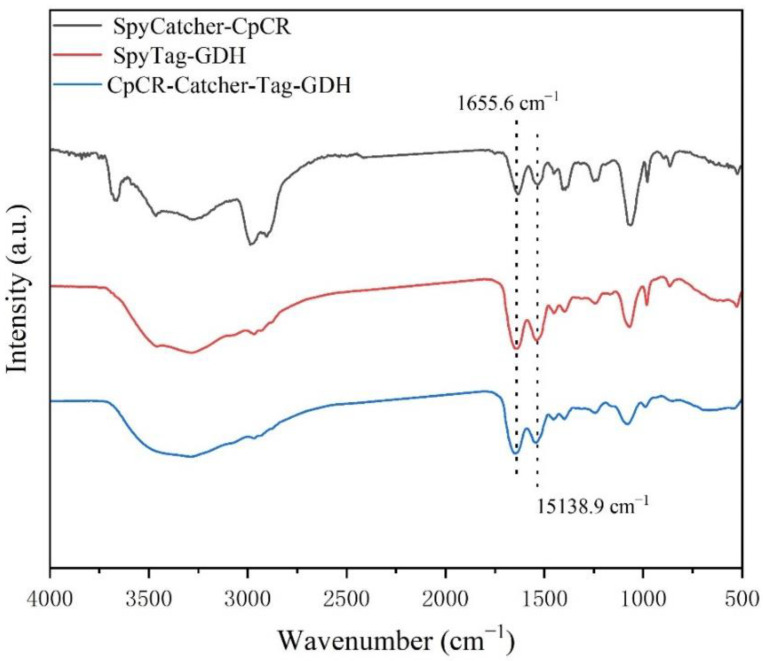
Infrared spectrum.

**Figure 19:**
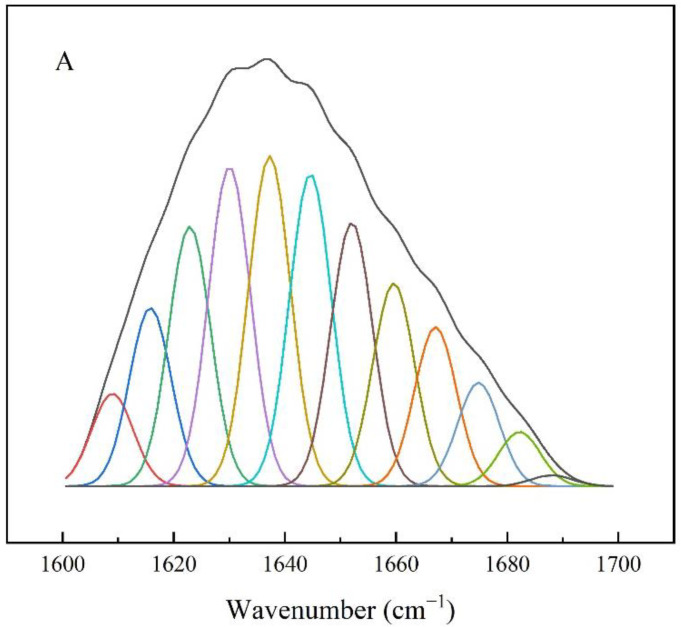

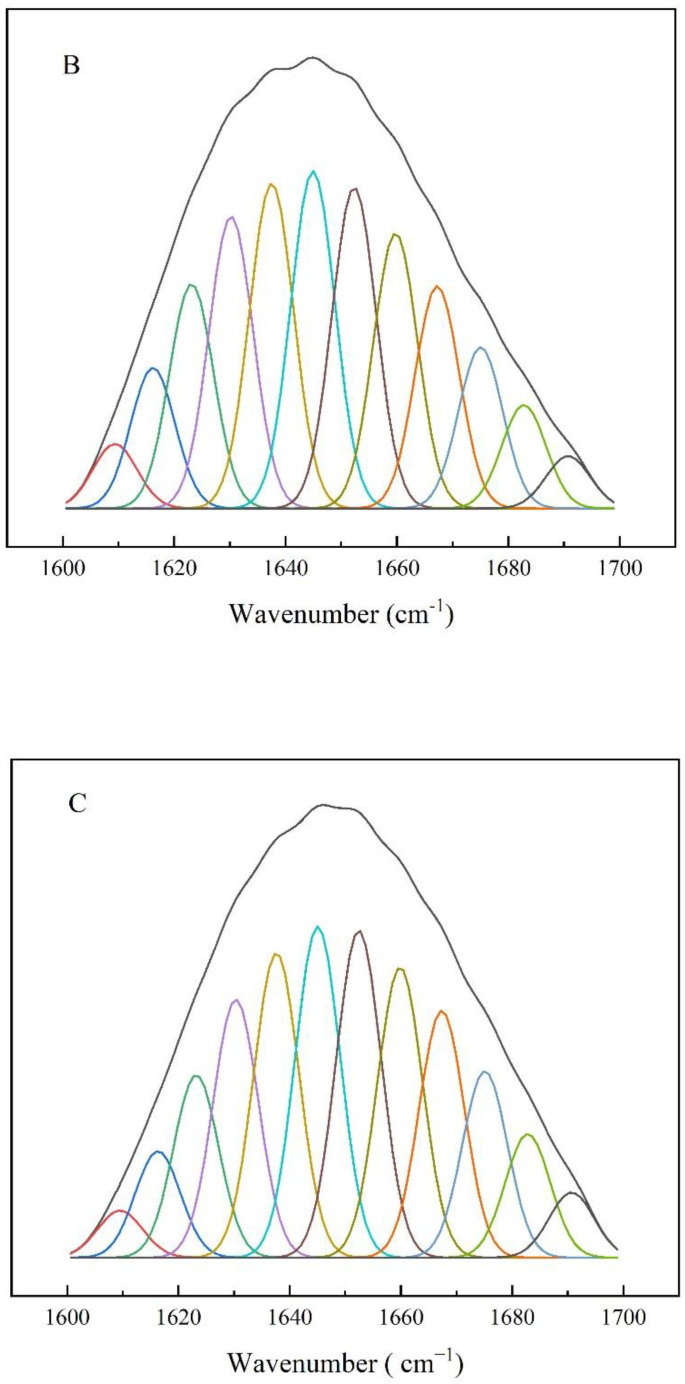
Fitting diagrams of amide Ⅰ band of different enzymes. (**A**) SpyCatcher-CpCR. (**B**) SpyTag-GDH. (**C**) CpCR-SpyCatcher-SpyTag-GDH.

The characteristic peak at 1655.6 cm^−1^ was attributed to the tensile vibration of C=O and the stretching and bending vibrations of N-H in the amide I band. In addition, the peak at 1538.9 cm^−1^ corresponds to the bending vibrations of the C-N and N–H bonds in the amide II band, indicating that SpyCatcher-CpCR and SpyTag-GDH were successfully assembled and cross-linked (Figure 18). As seen in Table 9, the α-helix content of the enzyme increased by 4.6% and the β-sheet content decreased by 15.21% after self-assembly compared with SpyCatcher-CpCR, and the α-helix content of the enzyme increased by 1.34% and the β-sheet content decreased by 5.12%. These conformational changes resulted in the increased catalytic performance of CpCR-SpyCatcher-SpyTag-GDH (Figure 19).

## 4. Conclusions

In conclusion, the self-assembled CpCR-SpyCatcher-SpyTag-GDH exhibited a higher catalytic efficiency than the unassembled enzyme. CpCR-SpyCatcher-SpyTag-GDH was obtained at a 1:1 molar ratio of SpyCatcher-CpCR and SpyTag-GDH after 3 h. Conversion reached 99% and the *ee*% of (*R*)-HPBE reached above 99% when 50 mM OBPE was converted by 25 mM CpCR-SpyCatcher-SpyTag-GDH at pH 7, 30 °C for 12 h. Moreover, the conversion achieved by CpCR-SpyCatcher-SpyTag-GDH was 1.6 times higher than that of the free enzymes.

The BESCs (CpCR-SpyCatcher-SpyTag-GDH) formed an ordered pore-like network structure compared with the unassembled enzymes. Compared with the free enzymes, the assembled structure increased the temperature and pH stability of CpCR-SpyCatcher-SpyTag-GDH. We constructed a supramolecular catalytic system based on SpyCatcher/SpyTag for CpCR and GDH, and applied it to the biosynthesis of (*R*)-HPBE. Using electron microscopy and other characterization techniques, CpCR-SpyCatcher-SpyTag-GDH showed a two-dimensional lamellar structure. Compared with the free bi-enzyme, this kind of macromolecules bi-enzyme self-assembled cluster not only had a more efficient catalytic efficiency, but also a better stability. The biocatalytic efficiency and stability of the cascade reaction of CpCR-SpyCatcher-SpyTag-GDH were improved compared with the free bi-enzyme system. Therefore, CpCR-SpyCatcher-SpyTag-GDH, a covalent protein complex, has great potential applications in synthetic biology.

## Figures and Tables

**Table 1:** Strains used in the experiment.

Strains and Plasmids	Application	Source
*Candida parapsilosis* ATCC 7330	Source of CpCR	ATCC
*E.coli* DH5α	Cloning host	Tsingke
*E.coli* BL21(DE3)	Expression host	Our laboratory
DH5α(pET28a-GDH)	Source of GDH	Gene synthesis
BL21 (pETDuet-CpCR)	BL21(DE3) harbouring (pETDuet-CpCR)	This study
BL21 (pETDuet-Catcher-CpCR)	BL21(DE3) harbouring (pETDuet- Catcher-CpCR)	This study
BL21 (pET28a-GDH)	BL21(DE3) harbouring (pET28a-GDH)	This study
BL21 (pET28a-Tag-GDH)	BL21(DE3) harbouring (pET28a-Tag-GDH)	This study

**Table 2:** Primers used in this study.

Primer Name	Primer Sequences (5′→3′)	Restriction Cite
CpCR-F1	AACTGCAGATGACTAAAGCAGTACCAGA	Pst І
CpCR-R1	CCGCTCGAGAGCTTTGAATGCTTTGT	Xho І
CpCR-R2	CCGCTCGAGTCAGTGGTGGTGGTGGTGGTGAGCTT	Xho І
Tag-C-F	cgggtccggaaggcggatccATGACTAAAGCAGTACCAGACAAGT	
Tag-C-R	tggtggtggtggtgctcgagAGCTTTGAATGCTTTGTCGAAATCA	
Cat-G-F	tggaggttccctgcagATGTATCCGGATCTGAAAGGTAAAG	
Cat-G-R	gtttctttaccagactcgagtcaGTGGTGGTGGTGGTGGTGACCACGACC	

**Table 3:** Plasmids constructed in this study.

Plasmids		
pET28a(+)	Expression vector	Our laboratory
pETDuet-1	Expression vector	Our laboratory
pETDuet-CpCR	Vector for carbonyl reductase expression	This study
pETDuet-Catcher-CpCR	Fusion expression vector carbonyl reductase and SpyCatcher	This study
pET28a-GDH	Vector for glucose dehydrogenase expression	This study
pET28a-Tag-GDH	Fusion expression vector glucose dehydrogenase and SpyTag	This study

**Table 4:** Protein sequences in this study.

Enzymes/Proteins	Sequences
CpCR	MTKAVPDKFQGFAVSDPKNWNRPKLASYERKQINPHDVVLKNEVCGLCYSDIHTLSAGWQPLQRDNLVVGHEIIGEVIAVGDEVTEFKVGDRVGIGAASSSCRSCQRCDSDNEQYCKQGAATYNSKDVRSNNYVTQGGYSSHSIADEKFVFAIPEDLPSSYGAPLMCAGITVFSPLIRNLGLDARGKNVGIIGIGGLGHLALQFANAMGANVTAFSRSSSKKEQAMKLGAHDFVATGEDKTWYKNYDDHFDFILNCASGIDGLNLSEYLSTLKVDKKFVSVGLPPSEDKFEVSPFTFLQQGASFGSSLLGSKTEVKEMLNLAAKHNVRPMIEEVPISEENCAKALDRCHAGDVRYRFVFTDFDKAFKA
GDH	MYPDLKGKVVAITGAASGLGKAMAIRFGKEQAKVVINYYSNKQDPNEVKEEVIKAGGEAVVVQGDVTKEEDVKNIVQTAIKEFGTLDIMINNAGLENPVPSHEMPLKDWDKVIGTNLTGAFLGSREAIKYFVENDIKGNVINMSSVHAFPWPLFVHYAASKGGIKLMTETLALEYAPKGIRVNNIGPGAINTPINAEKFADPKQKADVESMIPMGYIGEPEEIAAVAAWLASKEASYVTGITLFADGGMTQYPSFQAGRG
SpyCatcher	GAMVDTLSGLSSEQGQSGDMTIEEDSATHIKFSKRDEDGKELAGATMELRDSSGKTISTWISDGQVKDFYLYPGKYTFVETAAPDGYEVATAITFTVNEQGQVTVNGKATKGDAHI
Linker 1	GGGGSGGGGS
SpyTag	AHIVMVDAYKPTK
Linker 2	AGAGAGPEGAGAGAGPEGAGAGAGPEGAGAGAGPEGAGAGAGPEG

**Table 5:** Enzyme activity assay.

Enzymes	Enzyme Activity (U/mg)
CpCR	16.67 ± 0.38
SpyCatcher-CpCR	17.91 ± 0.42
SpyTag-CpCR	10.35 ± 0.82
GDH	10.95 + 0.47
SpyCatcher-GDH	10.57 ± 0.38
SpyTag-GDH	10.46 ± 0.62

**Table 6:** Assembly of BESCs with different molar ratios.

Modular Protein	Molar Ratio	Assembly Efficiency
2 µM SpyCatcher-CpCR:2 µM SpyTag-GDH	1:3	84.5%
	1:2	92.7%
	1:1	97.6%
	2:1	96.5%
	3:1	92.1%
2 µM SpyTag-CpCR:2 µM SpyCatcher- GDH	1:3	64.6%
	1:2	77.3%
	1:1	82.5%
	2:1	80.2%
	3:1	82.3%

Concentrated 10 times by ultrafiltration and measured concentration.

**Table 7:** Dock score.

Enzymes	CpCR	SpyCatcher-CpCR	SpyTag-CpCR	CpCR-SpyCatcher-SpyTag-GDH	CpCR-SpyTag-SpyCatcher-GDH
LibDockScore	86.76	90.78	79.79	83.68	76.78

**Table 8:** Kinetic parameters of the free enzyme and CpCR-SpyCatcher-SpyTag-GDH.

Rection Systems	Vmax (min)	Km (mM)	Kcat (s^−1^)	Kcat/Km (s^−1^·mM^−1^)
free enzyme	0.0176	23.94	1.76	0.074
CpCR-SpyCatcher-SpyTag-GDH	0.0212	21.75	2.12	0.097

**Table 9:** Secondary structure composition contents.

	β-Sheet/%	Random Coil/%	α-Helix/%	β-Turn/%
SpyCatcher-CpCR	51.61	13.66	20.40	14.33
SpyTag-GDH	41.55	13.39	23.66	21.40
CpCR-SpyCatcher-SpyTag-GDH	36.40	13.40	25.00	25.20

## Data Availability

All data generated or analyzed during this study are included in this article.
